# An E2F1-HOXB9 Transcriptional Circuit Is Associated with Breast Cancer Progression

**DOI:** 10.1371/journal.pone.0105285

**Published:** 2014-08-19

**Authors:** Aisulu Zhussupova, Tetsu Hayashida, Maiko Takahashi, Kazuhiro Miyao, Hiroshi Okazaki, Hiromitsu Jinno, Yuko Kitagawa

**Affiliations:** Department of Surgery, Keio University School of Medicine, Tokyo, Japan; Institut de Génomique Fonctionnelle de Lyon, France

## Abstract

Homeobox B9 (HOXB9), a member of the homeobox gene family, is overexpressed in breast cancer and promotes tumor progression and metastasis by stimulating epithelial-to-mesenchymal transition and angiogenesis within the tumor microenvironment. HOXB9 activates the TGFβ-ATM axis, leading to checkpoint activation and DNA repair, which engenders radioresistance in breast cancer cells. Despite detailed reports of the role of HOXB9 in breast cancer, the factors that regulate *HOXB9* transcription have not been extensively examined. Here we uncover an underlying mechanism that may suggest novel targeting strategies for breast cancer treatment. To identify a transcription factor binding site (TFBS) in the *HOXB9* promoter region, a dual luciferase reporter assay was conducted. Protein candidates that may directly attach to a TFBS of *HOXB9* were examined by Q-PCR, electrophoretic mobility shift assay (EMSA), chromatin immunoprecipitation (ChIP), and mutation analysis. A *HOXB9* promoter region from −404 to −392 was identified as TFBS, and E2F1 was a potential binding candidate in this region. The induction of *HOXB9* expression by E2F1 was observed by Q-PCR in several breast cancer cell lines overexpressing E2F1. The stimulatory effect of E2F1 on *HOXB9* transcription and its ability to bind the TFBS were confirmed by luciferase, EMSA and ChIP assay. Immunohistochemical analysis of 139 breast cancer tissue samples revealed a significant correlation between E2F1 and HOXB9 expression (p<0.001). Furthermore, a CDK4/6 inhibitor suppressed E2F1 expression and also reduced expression of *HOXB9* and its downstream target genes. Our in vitro analysis identified the TFBS of the *HOXB9* promoter region and suggested that E2F1 is a direct regulator of *HOXB9* expression; these data support the strong correlation we found between E2F1 and HOXB9 in clinical breast cancer samples. These results suggest that targeting the E2F1/HOXB9 axis may be a novel strategy for the control or prevention of cancer progression and metastasis.

## Introduction

Early diagnosis and treatment based on the molecular characteristics of breast tumors has significantly improved patients' survival over the past few decades [1]. However, the etiology of many cancers, and the signaling pathways that are required for tumor formation and metastasis remain poorly understood in many cases. Consequently, the identification of novel genes and genetic signatures has great potential in providing new approaches in clinical practice for controlling cancer progression.

*Homeobox (HOX)* genes are located in four clusters (A–D) on different chromosomes (7p15, 17p21, 12q13, and 2q3). HOX genes play important role as developmental regulators and likely to be influenced by various factors [2]. Several studies have showed the influence of retinoic acid [3], [4], [5] and steroid hormones in regulation of HOX genes [6]. HOX genes are deregulated in solid tumors, including those of colorectal cancer [7], lung adenocarcinoma [8], small cell lung cancer [9] and ovarian cancer [10]. In breast cancer, *HOXB7* and *HOXB13* are overexpressed [11], [12] and associated with increased cell proliferation. Correspondingly, differentiation and antagonism of these genes was recently shown to circumvent tamoxifen resistance [13].

*HOXB9* overexpression was identified in 43% of breast cancer tissues and strongly correlated with high tumor grade. This is likely due to its induction of TGF-β, ErbB ligands and several angiogenic factors, including VEGF, bFGF, IL-8, and ANGPTL-2; these factors contribute to increased cell motility, invasion, and angiogenesis [14], [15]. The comparison between HOXB9-positive and -negative tumors of breast cancer patients revealed a considerable difference in disease-free and overall survival rate [16]. Moreover, breast cancer cells overexpressing HOXB9 are resistant to ionizing radiation due to an enhanced DNA damage response. The ability of HOXB9 to modulate the damage response requires baseline ATM activity before irradiation and also involves epithelial-to-mesenchymal transition induced by TGF-β, a HOXB9 transcriptional target [17]. These data reveal the impact of the HOXB9-TGFβ-ATM axis on checkpoint activation and DNA repair. HOXB9 was also reported as promoter of lung adenocarcinoma metastasis and multiorgan metastatic progression of lung cancer due to its activation of the WNT/TCF pathway [18], as well as promoter of metastasis in colon cancer and potential biomarker for bevacizumab treatment [19], [20]. On the basis of these background information, we hypothesized that to target this coordinated program orchestrated by HOXB9 may be required for effective inhibition of tumor progression, and focused on the mechanisms of HOXB9 transcription. In this study, we identified upstream regulatory elements that are critical for *HOXB9* transcription and E2F1 was suggested as a protein candidate to bind the region. Moreover, we demonstrated that PD-0332991, a selective inhibitor of CDK4/6, concomitantly inactivates E2F1 and downregulates *HOXB9* expression. Together these data provide a clear mechanistic link between an oncogene and a tumor promoter, and suggest that disruption of this link should be considered as a future anticancer strategy.

## Materials and Methods

### Cell culture and transfection of reporter plasmids

The human breast cancer cell lines MCF7 and MDA-MB-231 were grown in DMEM medium supplemented with 10% heat-inactivated fetal bovine serum (FBS, Life Technologies, Carlsbad, CA), penicillin (50 U/mL) and streptomycin (50 µg/mL). Transfection was performed using FuGENE 6 (Roche Diagnostics, Indianapolis, IN) according to the manufacturer's protocol. Cells were transfected in Opti-MEM reduced serum media (Life Technologies).

### Bioinformatic analysis and cloning of the *HOXB9* promoter region

Genomic DNA of the full length *HOXB9* promoter region, from the 5' flanking −2,989 upstream region sequence to the +246 downstream region, and from the transcriptional start site of the *HOXB9* gene, was amplified using genomic DNA as a template and PCR primer with incorporated *Xho*I (HOXB9-2898) and *Hind*III (HOXB9+246R).

The PCR product after digestion with *Xho*I and *Hind*III was inserted into the pGL3-Basic vector (Promega, Madison, WI), and the entire length of the inserted PCR product was sequenced using an ABI Prism 310 machine (Applied Biosystems, Foster city, CA).

### Construction of recombinant luciferase reporter plasmids with the full-length *HOXB9* promoter region and its derivatives

First, HOXB9 primer from -2898 to -204 bps was subcloned into the *Xho*I-*Hind*III site of the pGL3-Basic vector (Promega), and the plasmid obtained was designated pGL3-2898. Several deletion variants designated pGL3-1865, pGL3-1415, pGL3-920, pGL3-439, pGL3-427, pGL3-417, pGL3-412, pGL3-404, pGL3-392, pGL3-296, pGL3-258, pGL3-204, and pGL3+1 were generated using primer sets of the forward primer with an incorporated *Xho*I site or the reverse primer with an incorporated *Hind*III site as described above. The number of each deletion variant represents the position towards 5′ flanking site.

The mutant DNA sequences of the *HOXB9* promoter region were generated using a QuikChange Site-Directed Mutagenesis kit (Stratagene, La Jolla, CA) and inserted into pGL3-Basic. Two types of mutations were constructed and designated pGL3–HOXB9–WT and pGL3–HOXB9–DT. For pGL3–HOXB9–WT construction, the GGC sequence was changed to ATT.

### Dual- luciferase reporter assay

Dual-luciferase reporter assays with the constructed reporter plasmids were carried out according to the manufacturer's instructions (Promega, Madison, WI). Transfections were carried out on 24-well plates, and the reporter assays were carried out on 96-well plates. Twenty hours after transfection, the cell culture medium was replaced with fresh complete growth medium, and the cells were incubated for another 24 h. The activity of firefly (*Photinus pyralis*) and Renilla (*Renilla reniformi*) luciferases in cell lysates was measured sequentially with a Microlumat *Plus* 96-well plate luminometer (Berthold technologies, Bad Wildbad, Germany). Data are representative of more than three independent experiments.

### RT-PCR

Total RNA was prepared from MCF7, MCF7/E2F1, MCF10A, MDA-MB-231 and BT-549 cells using RNeasy Mini Kit (Qiagen, Venlo, Limburg, Netherlands) according to the manufacturer's instructions. The resultant first-strand cDNA was synthesized using a High Capacity RNA-to-cDNA kit to examine the expression levels of genes of interest. Quantitative PCR (Q-PCR) was performed using SYBR Green (Applied Biosystems) on an ABI Prism 7500 apparatus (Applied Biosystems) in three independent experiments. *GAPDH* was used as an internal control. The comparative threshold (ΔCt) method was used. The primer sequences are provided in Table S1.

### Small interfering RNA (siRNA) transfection

The E2F1 small interfering RNA (siRNA) and control, non-targeted siRNA, were purchased from Dharmacon (GE Healthcare, Little Chalfont, UK) and transfected to BT-549 cells. The transfection was performed by Lipofectamine 2000 (Invitrogen) reagent into BT-549 cells following the manufacturer's instructions. The E2F1 siRNA knockdown expression after the transfection of siRNA was determined by Q-PCR analysis at 72 h.

### Chromatin immunoprecipitation assay

MCF7 cells overexpressing exogenous E2F1 were cross-linked with 1% formaldehyde in medium for 15 min at 37°C. Cells were then washed with ice-cold PBS and resuspended in 200 µL of SDS lysis buffer containing a protease inhibitor cocktail (Roche, Basel, Switzerland). The cell suspension was sonicated to produce DNA fragments with an average length of 200–600 nt and then pre-cleared with protein A-agarose beads for 30 min at 4°C. The beads were removed by a brief centrifugation and the chromatin solution was immunoprecipitated with normal rabbit serum (NRS) or with anti-E2F1 antibody (Cell Signaling Technology, Boston, MA) overnight at 4°C, followed by incubation with protein A-agarose beads for an additional 1 h at 4°C. The immune complex was eluted with 100 µL of elution buffer (1% SDS and 0.1 M NaHCO_3_), and formaldehyde cross-linking was reversed by heating at 65°C for 4 h. Genomic DNA was purified from the immunoprecipitates and analyzed by standard PCR. The specific primers used in this study were as described: TFBS of *HOXB9* 5'-TCTACAGCCTGCGTCCCTCCAA-3' (sense) and 5'-ATGTGCTATCACGTCAGGGCTCC-3' (antisense), non-critical region *HOXB9* 5'-TGATCCGCAGCCTTCTACAAGGC-3' (sense) and 5'-GGTTAGGCTCAGGGGTAGATTGG-3' (antisense).

### Electrophoretic mobility shift assay (EMSA)

Nuclear proteins were prepared from T47D cells cultured for 12 h utilizing NE-PER Nuclear and Cytoplasmic Extraction Reagents (Thermo Scientific, Waltham, MA). The *HOXB9* promoter sequence utilized as an oligonucleotide probe was 5'-GGGGGACCGGGCGGCCGGTAGCTG-3'. The 5' end of the oligonucleotide was labeled with biotin, and a complimentary oligonucleotide was annealed to generate double-stranded fragments. EMSA was performed using the Thermo Scientific LightShift Chemiluminescent RNA EMSA Kit, a non-radioactive (biotin labeled) gel shift assay, according to the manufacturer's protocol.

Anti-E2F1 antibody (Cell Signaling Technology, Boston, MA) was used at dilutions of 1∶33, 1∶100 and 1∶200. To confirm the specificity of E2F1 binding to the *HOXB9* promoter sequence, we serially diluted the anti-E2F1 antibody before incubation with nuclear extract prior to EMSA.

The sequence of the non-specific probe was 5′- TTTTTATTTTGTTTTAATCTGAAA-3′.

### Tissue samples

A panel of 232 breast tumors presented chronologically between January 2004 and January 2005 at Keio University Hospital was identified in the hospital breast cancer database. Of 155 consecutive, invasive ductal carcinoma patients who were diagnosed as having stage I, II, or III disease and who had not received any treatment before surgery, 139 paraffin-embedded tissue specimens were available for immunohistochemical (IHC) assessment [16]. Written informed consent was taken and the study was approved by the ethics committee of the School of Medicine of Keio University.

### Western blot and immunohistochemistry

For Western blots (WB), protein samples were quantified and 60 µg fractionated on 4–15%, 10-well comb Mini-PROTEAN TGX gels (Bio-Rad Laboratories, Hercules, CA), electrophoretically transfered onto TransBlot Turbo Mini-size PVDF Membrane (Bio-Rad), blocjed in Amersham ECL Blocking Agent (GE Healthcare, Buckinghamshire, UK) in Tris-buffered saline and 0.1% Tween (30–60 min, room temperature) and exposed to the primary antibody overnight at 4°C. Membranes were exposed to the manufacturer- recommended dilution of the appropriate secondary antibody anti-rabbit NA934V (GE Healthcare) and anti-mouse NA931V (GE Healthcare). Proteins were visualized by Luminata Forte Western HRP Solution (Millipore, Bilerica, MA). Western blot primary antibodies: anti-HOXB9 (A-21) 1∶600 (Santa Cruz Biotechnology, Inc., Santa Cruz, CA), anti-E2F1 (EPR3818(2)) 1∶800 (Abcam, Cambridge, England) and anti-GAPDH (0411) 1∶2000 (Santa Cruz Biotechnology).

Paraffin-embedded sections were cut into 4-µm-thick serial tissue sections, mounted onto slides, dewaxed in xylene, and rehydrated in alcohol. Endogenous peroxidase activity was then blocked with peroxidase blocking reagent (0.03% hydrogen peroxide containing sodium azide; Dako, Carpinteria, CA) for 10 min. Afterwards, the sections were autoclaved in target retrieval solution (pH 9.0; Dako) at 121°C for 10 min. Samples were incubated with primary antibody to E2F1 (clone KH95, Santa Cruz Biotechnology) at a dilution of 1∶50 at 4°C for 12 h, followed by a secondary antibody (Dako EnVision+, HRP. K4001) for 30 min. Diaminobenzidine tetrahydrochloride (DAB) substrate buffer (Substrate buffer solution, pH 7.5; Dako) mixed with DAB chromogen (Diaminobenzidine chromogen solution; Dako) was added for 5 min. Between these steps, the slides were rinsed for 5 min in phosphate buffered saline six times. Sections were then counterstained with hematoxylin, dehydrated, and mounted. E2F1 expression was independently evaluated by two authors (T.H. and A.Zh.), both of whom were blinded to the clinicopathologic data. We followed the methodical recommendation described in previous research [21]; staining intensity was scored as weak or strong, and both were scored as being E2F1 positive. The clinicopathologic variables were compared by Fisher's exact test, *X^2^*- test, or logistic regression where appropriate.

### CDK4/6 inhibitor treatment

BT-549 and MCF7 cells were treated with CDK4/6 inhibitor (PD-0332991) (Active Biochem) at a final concentration of 5 µM for 48 h. Treated cells were routinely grown in DMEM supplemented with 10% heat-inactivated FBS, penicillin (50 U/mL), and streptomycin (50 µg/mL).

## Results

### Promoter analysis

To find direct inducers of *HOXB9* expression, we investigated the *HOXB9* promoter region to determine potential transcription factor-binding sites (TFBS).

**Figure 1:**
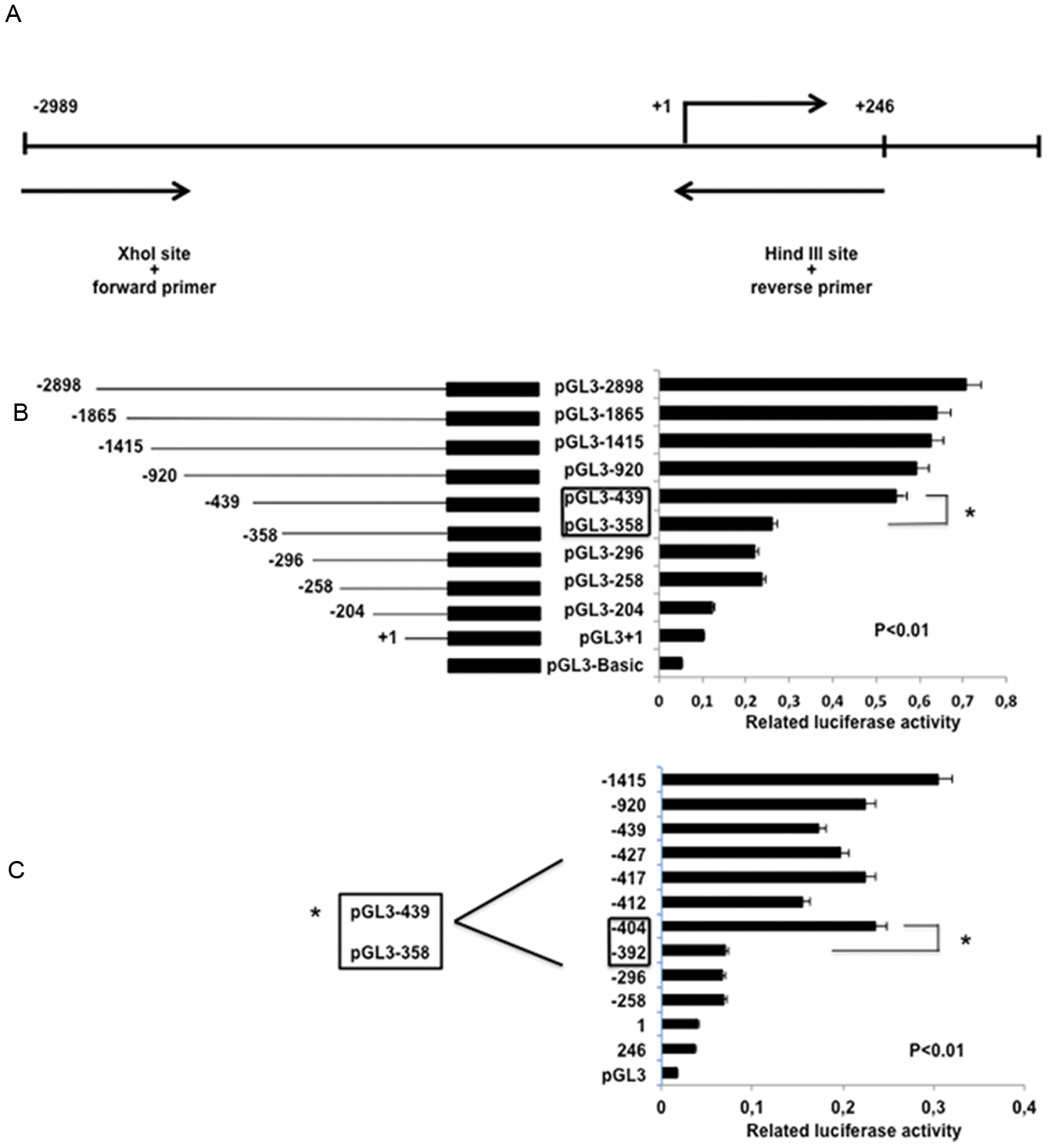
Identification of transcription factor binding site (TFBS) of *HOXB9* promoter. (**A**) *HOXB9* promoter region and primer sets used for cloning. (**B**) Reporter plasmids with the *HOXB9* promoter region and deletion variants were constructed by inserting the PCR product of the putative HOXB9 promoter region. The numbers of the left side of graph indicate the full length of HOXB9 promoter and deletion variants, the right side shows transcriptional activity of each variant in MDA-MB231 cells. The difference in relative luciferase activity between pGL3-439 and pGL3-358 constructs was recorded. (*p<0.01) (**C**) Additional deletion plasmids between bps −439 and −358 of the promoter were constructed to identify the exact position of TFBS and gap of gene activity in that region was recorded each time when assay was repeated. The difference in relative luciferase activity between pGL3-404 and pGL3-392 constructs was significant (*p<0.01).

The *HOXB9* long 5′ flanking region from −2,989 to +246 (the transcription start site was designated as +1) was obtained by PCR amplification of genomic DNA from the human breast cancer cell line MCF7 (Figure 1A). Promoterless luciferase vector pGL3 was used for fragment subcloning, and the putative promoter activity was measured by a dual-luciferase assay. The deletion variants of the *HOXB9* promoter region yielded different basal luciferase expression levels. pGL3-439 exhibited an approximately 2-fold increase in relative luciferase activity in comparison with that of pGL3-358 (p<0.01, Figure 1B)

Further detailed mapping of activity in this promoter region displayed the gap of transcriptional activity in the region between pGL3-404 and pGL3-392, and the difference was recorded each time when the assay was repeated four times. The difference in relative luciferase activity between pGL3-404 and pGL3-392 constructs was significant (p<0.01, Figure 1C).

### Transcription factors regulating *HOXB9* expression

The data obtained suggested that the sequence harboring a TFBS in the *HOXB9* promoter was between −404 and −392 bps (CCGGGCGGCCGG). Further experiments were performed to check for candidate transcription factors that could bind to this region of the promoter and could induce *HOXB9* transcription.

The *HOXB9* promoter sequence from −404 to −392 was subjected to computational analysis by the PROMO 3.0 software program. The program identified several candidate transcription factors: E2F1, SP1, TP53, and PAX5, which may directly bind to the *HOXB9* promoter sequence; all of the candidate transcription factors are known to be associated with cancer progression.

**Figure 2:**
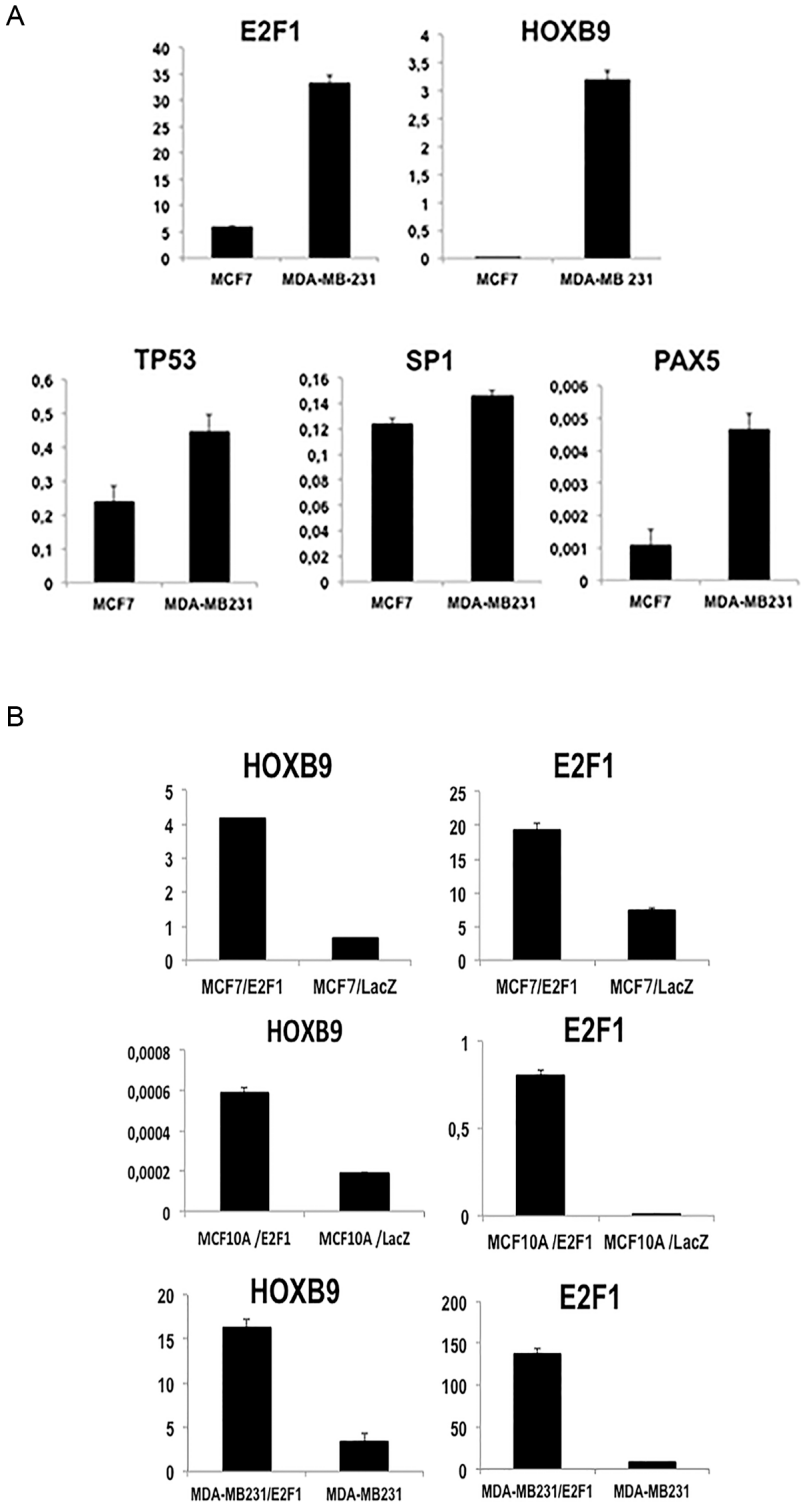
E2F1 as a possible candidate for regulating *HOXB9* expression. Bioinformatic analysis of transcription factor binding sites (TFBS) of the *HOXB9* region suggested several candidates as transcription factors: E2F1, PAX5, TP53, and SP1. (**A**) The mRNA expression of such candidates in MCF7 (HOXB9 expression low) and MDA-MB-231 (HOXB9 expression high) indicated the possible binding candidate by Q-PCR analysis. (**B**) The mRNA level in comparison with GAPDH of *HOXB9* and *E2F1* in MCF10A, MCF7, and MDA-MB-231 either with or without exogenous E2F1 displayed the positive correlation.

The mRNA expression of all candidates, in comparision with GAPDH, was analyzed in basal type (MDA-MB-231) and hormone positive (HR(+)), HER2 negative (HER2 (−)) type MCF7 breast cancer cell lines. The analysis revealed that E2F1 was the strongest candidate, since its expression was much higher in MDA-MB-231 cells, which are already known to overexpress HOXB9 [14], than in MCF7 cells (Figure 2A).

**Figure 3:**
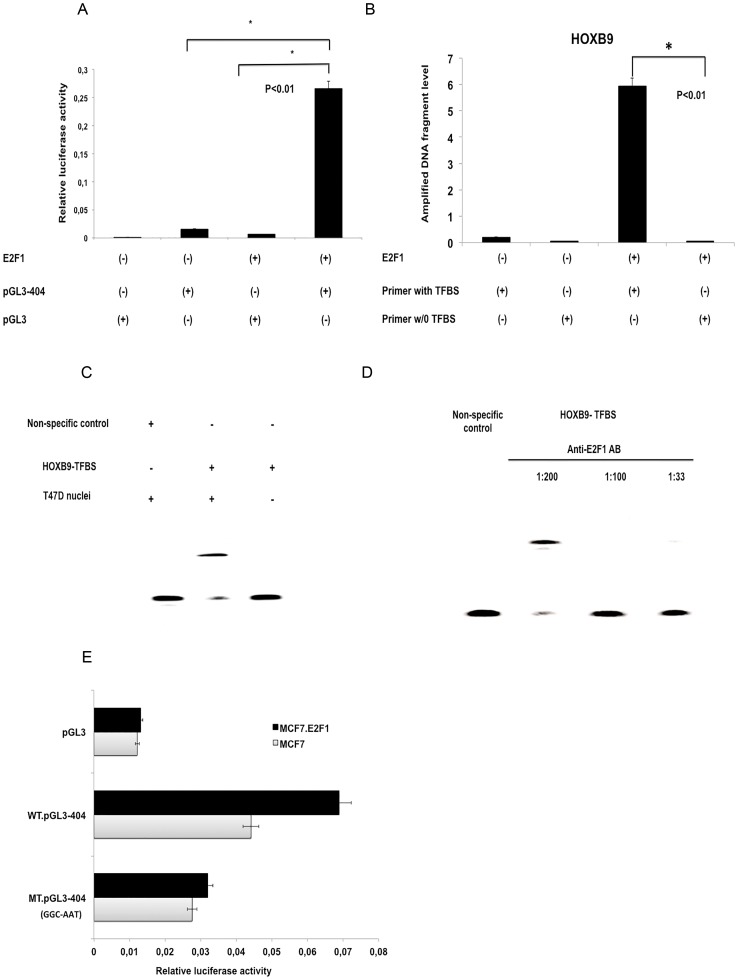
*HOXB9* is a transcriptional target of E2F1. (**A**) A dual luciferase assay with plasmid including the *HOXB9* TFBS (pGL3-404) was analyzed in MCF7 wild type and MCF7 cells overexpressing E2F1 to indicate transcriptional activity. MCF7 cells without E2F1 overexpression and the pGL3 vector plasmid were used as negative control. (**B**) Genomic DNA extracted from MCF7 either with or without exogenous E2F1 overexpression was precipitated with anti-E2F1 antibody for ChIP analysis. The amplified DNA fragment level was confirmed by Q-PCR with a primer set containing TFBS of *HOXB9*. Another primer set that does not include *HOXB9* TFBS fragment was used as a negative control. (**C**) EMSA was performed with protein extract from T47D nuclei and a biotinylated probe coding the *HOXB9* promoter sequence from −404 to −392 bp (HOXB9-TFBS). A non-specific probe not containing the TFBS was used as a negative control. (**D**) EMSA was performed after a 40-min incubation with T47D nuclei extract in several concentrations of anti-E2F1 antibody (1∶200, 1∶100, and 1∶33). (**E**) Mutation variants of the *HOXB9* TFBS, in which the GGC sequence was changed to ATT, cloned into pGL3 plasmid, and analyzed using a dual luciferase assay in MCF7 either with or without exogenous E2F1. The pGL3 vector plasmid was used as negative control. WT, wild type; MT, mutation variant.

In order to further investigate the relationship between HOXB9 and E2F1, we established E2F1-overexpressing and LacZ control cell lines from MDA-MB-231, MCF7 and MCF10A cells. All the E2F1-overexpressing lines displayed higher *HOXB9* expression in comparison to the control (Figure 2B). We subsequently performed a luciferase assay to verify the activity of the pGL3-404 plasmid, which contained the putative TFBS of *HOXB9*. MCF7-E2F1 cells elicited higher luciferase activity compared to parental MCF7 (p<0.01, Figure 3A), suggesting that E2F1 can promote *HOXB9* transcription.

### Direct induction of *HOXB9* by E2F1

Next, experiments were designed to evaluate whether E2F1 protein might directly bind to the TFBS of the *HOXB9* promoter. For chromatin immunoprecipitation (ChIP) assay, one primer pair was designed to target the region bound by E2F1 (*HOXB9* promoter sequence from bps −404 to −392), whereas another primer pair was designed to target a proximal site in the HOXB9 promoter that is not predicted as an E2F1 binding site. Q-PCR demonstrated the primer set with TFBS has significantly higher expression in comparison with the control (p<0.01), suggesting that E2F1 could directly bind to the TFBS of *HOXB9* (Figure 3B).

EMSA was also performed to confirm E2F1 binding using a biotinylated probe encoding the *HOXB9* promoter sequence from −404 to −392. For this experiment, nuclei extracts from T47D cells were used, since the concentration of E2F1 protein in the nuclei of T47D is higher than the other breast cancer cell lines. A clear shift in the migration of the band of biotinylated probe was obtained, which represents E2F1 binding to the TFBS of the *HOXB9* promoter. On the other hand, neither biotinylated probe without T47D nuclei extract nor non-specific probe yielded a band (Figure 3C).

To confirm the specificity of E2F1 binding to the *HOXB9* promoter sequence, we serially diluted the anti-E2F1 antibody before incubation with nuclear extract prior to EMSA. The lowest concentration (1∶200) of E2F1 antibody still produced a bandshift, which disappeared while E2F1 concentration was increased, proving the specificity of antibody to E2F1 (Figure 3D).

A mutated variant of the *HOXB9* promoter sequence from −404 to −392 (pGL3-404-MT) was created, and luciferase activity was compared to pGL3-404 in MCF7 wild type and MCF7 cells overexpressing E2F1. The relative luciferase activity of the mutated plasmid decreased by almost 50% in comparison to pGL3-404 (Figure 3E). Together, these results demonstrate that E2F1 protein can directly bind to the TFBS of *HOXB9*.

### E2F1 regulates HOXB9 protein expression in breast cancer

**Figure 4:**
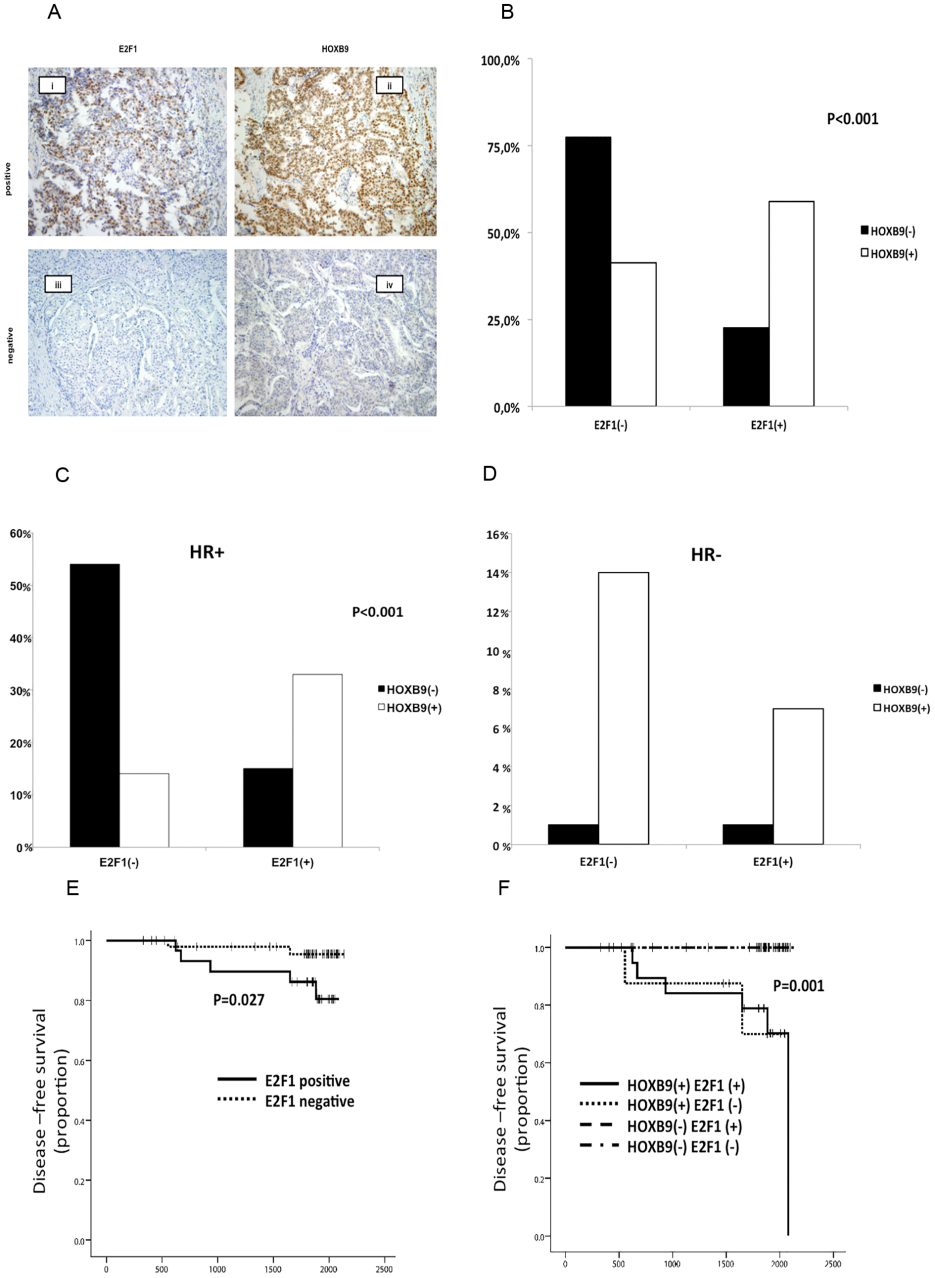
Correlation between E2F1 and HOXB9 protein expression in breast cancer tissues. (**A**) A cohort of 139 breast cancer clinical samples stained with anti-E2F1 antibody was interrogated for HOXB9 expression. i- E2F1 positive, ii- HOXB9 positive, iii- E2F1 negative, iv- HOXB9 negative staining (original magnification, 100×). (**B**) The cross-link results of 139 breast cancer clinical samples to evaluate the correlation between E2F1 and HOXB9 staining. (**C,D**) The data was analyzed by hormone receptor status. (**E**) Kaplan–Meier plots of clinical outcomes of HR(+)/HER2(−) type breast cancer patients by E2F1 expression. (**F**) Kaplan–Meier plots of breast cancer patients' clinical outcomes by combination of HOXB9 and E2F1 expression. All p values were calculated using the log rank test.

In our previous study, we examined 141 breast cancer tissues for HOXB9 expression by immunohistochemistry [16]. We used exactly the same cohort of tissue samples to stain for E2F1 expression. We considered E2F1 staining as positive when >10% of the cancer cell nuclei showed positive immunostaining, as recommended in a previous study [21] (Figure 4A).

The procedure revealed a significant correlation between E2F1 and HOXB9 staining (p<0.001, Table 1, Figure 4B) and this positive correlation was observed only in HR(+) group (Figure 4C, 4D). More than half of HR(+)/HER2(−) breast cancer tissues were negative for both HOXB9 and E2F1. A previous study revealed that HOXB9 positive staining was strongly associated with triple-negative and HER2 subtypes [16]; however, E2F1 was not related to these subtypes in the present study (Table 2). Survival analysis showed that E2F1 is a significant prognostic factor only among the HR(+)/HER2(−) breast cancer patients (Log-rank test; P = 0.027). Patients that were either positive for only HOXB9, or were HOXB9 and E2F1 double-positive, had a worse prognosis than double negative patients (Figure 4E,4F Table 2).

**Table 1:** The correlation of E2F1 and HOXB9 in IHC staining.

HOXB9	E2F1 (%)		Total (%)	P value
	Negative	Positive		
Negative	55	16	71	P<0.001
	(77.5)	(22.5)	(100)	
Positive	28	40	68	
	(41.2)	(58.8)	(100)	

**Table 2:** HOXB9/E2F1 staining and breast cancer subtypes.

	Subtypes (%)				All
	HR(+), Her2 (−)	HR(+), Her2 (+)	HR(−), Her2(+)	Triple neg.	
HOXB9(−), E2F1(−)	44	11	0	1	56
	(53.7)	(32.4)	(0.0)	(10)	(40.3)
HOXB9(+), E2F1(−)	8	5	9	5	27
	(9.8)	(14.7)	(69.2)	(50)	(19.4)
HOXB9(−), E2F1(+)	10	4	1	0	15
	(12.2)	(11.8)	(7.7)	(0.0)	(10.8)
HOXB9(+), E2F1(+)	20	14	3	4	41
	(24.4)	(41.2)	(23.1)	(40)	(29.5)
All	82	34	13	10	139
	(100)	(100)	(100)	(100)	(100)

HR: Hormone receptor.

HER2: Human Epidermal growth factor Receptor 2.

Triple neg: Triple negative.

### CDK4/6 inhibitor decreases E2F1-dependent HOXB9 expression

**Figure 5:**
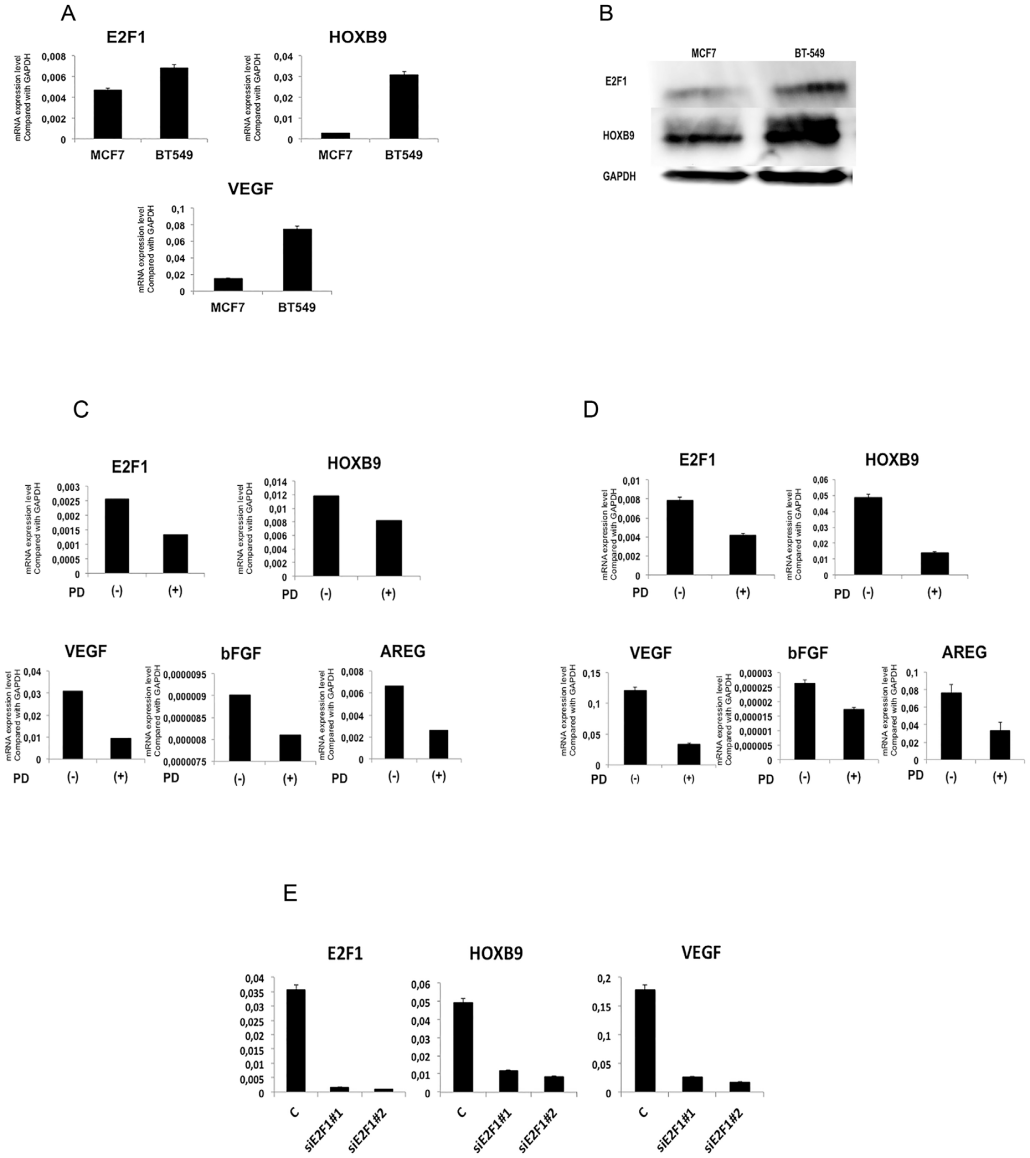
CDK4/6 inhibitor decreases *HOXB9* expression by E2F1. *HOXB9, E2F1* and *VEGF* expression in BT549 and MCF7 cell-lines was analyzed (**A**) by Q-PCR. (**B**) by Immunoblot analysis. GAPDH was used as a loading control. (**C**) MCF7 and (D) BT-549 cells were treated with PD-0332991 at a concentration of 5 µM for 48 h. Q-PCR analysis was used to determine the mRNA expression of *E2F1*, *HOXB9*, and its target genes (*VEGF*, *bFGF*, and *AREG*) in both cell-lines. (**E**) The expression of HOXB9 and VEGF was assessed in BT-549 cells in which E2F1 expression was knocked down with siE2F1. Non-targeted siRNA was used as negative control. Two different siRNA were used to avoid off-target effect.

An *in vitro* study in mantle cell lymphoma revealed that the CDK4/6 inhibitor, PD-0332991, suppressed E2F1 expression [22]. We thus investigated whether PD-0332991 also inhibits E2F1 expression in BT-549 breast cancer cells, which has high expression of endogenous HOXB9 and E2F1, compare to the control (MCF7 cells), (Figure 5 A,B) and whether this treatment would affect HOXB9 expression. Indeed, PD-0332991 treatment reduced expression of E2F1 and HOXB9 in both cell-lines (Figure 5C, 5D). The expression of the angiogenic factors VEGF, bFGF, and the ErbB ligand amphiregulin, which are known to be direct or indirect targets of HOXB9 [14] was also reduced. Additionally, the expression of HOXB9 and VEGF was also reduced in BT-549 cells in which E2F1 expression was knocked down with siE2F1 (Figure 5E). These results demonstrate that suppression of E2F1 by PD-0332991 leads to reduce expression of HOXB9 and its downstream targets.

## Discussion

In the present study, we demonstrate that the region from −404 to −392 of the *HOXB9* promoter plays an important role in *HOXB9* transcription, and that E2F1 is a strong candidate for a *HOXB9* regulatory transcription factor.

E2F1 is a member of the E2F transcription factor family and plays a key role in the G1 to S-phase transition by integrating numerous upstream signals, thereby determining whether cells should advance through the cell cycle or die via apoptosis [23]. E2F1 induces the expression of a wide spectrum of genes implicated in DNA synthesis, repair, cell-cycle control, and apoptosis. The transcriptional activity of E2F1 is negatively regulated by the pRb ‘pocket’ protein, which ‘masks’ its transactivation domain [24], [25], [26].

E2F1 is overexpressed in solid cancers, including non-small cell lung carcinoma, ovarian, prostate, and bladder cancers, colon cancer, and other digestive system malignances, and is associated with worse patient prognosis [27], [28], [29], [30], [31], [32], [33], [34], [35], [36]. In breast carcinomas, E2F1 expression is correlated with proliferation [29] and the poor survival of lymph node-positive breast cancer patients treated with fluorouracil, doxorubicin, and cyclophosphamide [37].

Further investigations using ChIP, luciferase, and EMSA technologies confirmed the *HOXB9* binding ability of E2F1. HOXB9 is also associated with a worse clinical prognosis in breast cancer patients. [16] In this study, IHC staining of 139 breast cancer patients' tissues was performed to assess the connection between E2F1 and HOXB9 in clinical samples, and E2F1 positivity and negativity was found to associate significantly (p<0.001) with HOXB9 staining, which was expected from our *in vitro* study.

In this cohort, more than half of HR(+)/HER2(−) and breast cancer tissues were negative for both HOXB9 and E2F1. In this subtype, E2F1-positive patients had a significantly worse clinical outcome, consistent with previous studies [37], [38]. HER2(+) and triple negative breast cancer was strongly associated with HOXB9 staining [16], but not with E2F1 reactivity. When a combined analysis of HOXB9 and E2F1 was conducted, HOXB9 positivity was the only factor that predicted a worse clinical outcome, and, in this case, E2F1 status did not have any further effect on prognosis. This result suggests that HOXB9 is likely regulated not only by E2F1, but also by the other transcription factors. Indeed, our *in silico* promoter sequence analysis identified a putative TFBS for SP1, TP53, and PAX5 and Ansari et al. reported that HOXB9 promoter contains several estrogen-response elements, being estrogen-responsive gene [39].

Combination treatment of the oral selective CDK4/6 inhibitor, PD-0332991, and letrozole was recently shown to substantially improve progression-free survival in patients with ER-positive breast cancer [40]. PD-0332991 was synergistic with tamoxifen and trastuzumab in ER(+) and HER2(−) amplified cell lines, and enhanced sensitivity to tamoxifen in cells with conditioned resistance to ER blockade [38]. Furthermore, PD-0332991 was also shown to downregulate E2F1 in mantle cell lymphoma [22]. In this study, PD-0332991 treatment decreased expression of E2F1 and reduced HOXB9 and its target genes in MCF7 breast carcinoma cells. Treatment with PD-0332991 causes cell cycle arrest through CDK4/6 inhibition, leading to tumor suppression [41]. However, our data suggest that this inhibitor may also act via inhibition of the E2F1-HOXB9 axis. Further investigation of PD-0332991 as a suppressor of HOXB9 expression in a clinical setting may provide a novel strategy to control breast cancer progression and prolong patients' survival.

## Supporting Information

Table S1 **Primers used for semi quantitative RT-PCR.**
(DOCX)
