# Supplementary material for: An E2F1-HOXB9 Transcriptional Circuit Is Associated with Breast Cancer Progression
Source: PLoS One. 2014 Aug 19;9(8):e105285. doi: 10.1371/journal.pone.0105285 (PMC4138122; doi:10.1371/journal.pone.0105285)
Supplement: Table S1 — Primers used for semi quantitative RT-PCR. (DOCX) [file pone.0105285.s001.docx]

**Supplement data.**

**Table S1. Primers used for semi quantitative RT-PCR**

| **Primer sequence** | | |
| --- | --- | --- |
| **Gene** | **Sense** | **Antisense** |
|  |  |  |
|  |  |  |
| HOXB9 | 5’-CCGGCTACGGGGACAATAA-3’ | 5’-GGTGTAGGGACAGCGCTTTTT-3’ |
| E2F1 | 5’-ACCATCAGTACCTGGCCGAGAG-3’ | 5’-TTGGTGGTCAGATTCAGTGAGGTC-3’ |
| TP53 | 5’-CCTCAACAAGATGTTTTGCCAACTG-3’ | 5’-GAGTCTTCCAGTGTGATGATGGTGAC-3’ |
| PAX5 | 5’-TCCCAGCTTCCAGTCACAG-3 | 5’-GGATGCCGCTGATGGAGTA-3’ |
| SP1 | 5’-CAGGCCCTCCAAGCAGGACC-’ | 5’-GGCCCCACTGTTGGTGTGTCCG-3’ |
| VEGF | 5’-AGAAGGAGGAGGGCAGAATC -3’ | 5’-TGG CTT GAA GAT GTA CTC GAT CTC -3’ |
| bFGF | 5’-TTCTTCCTGCGCATCCAC -3’ | 5’-CGGTTAGCACACACTCCTTTGAT -3’ |
| AREG | 5’-TACTCGGCTCAGGCCATTA -3’ | 5’-GAAATCTCACTCCCTGAAGACATC-3’ |
| GAPDH | 5’-ATCATCCCTGCCTCTACTGG-3’ | 5’-TTTCTAGACGGCAGGTCAGGT-3’ |
